# Microstructure, Mechanical Properties at Room Temperature and High Temperature of Near-α Titanium Alloys Fabricated by Spark Plasma Sintering

**DOI:** 10.3390/nano15040293

**Published:** 2025-02-14

**Authors:** Qiang Wang, Zhaohui Zhang, Xiaotong Jia, Yangyu He, Jinzhao Zhou, Yuanhao Sun, Xingwang Cheng

**Affiliations:** 1 School of Materials Science and Engineering, Beijing Institute of Technology, Beijing 100081, China; 3120225558@bit.edu.cn (Q.W.); 7520240147@bit.edu.cn (X.J.); 3120215537@bit.edu.cn (Y.H.); 3220235355@bit.edu.cn (J.Z.); 3120221082@bit.edu.cn (Y.S.); 2 Tangshan Key Laboratory of High-Performance Metals and Ceramics, Tangshan Research Institute BIT, Tangshan 063000, China

**Keywords:** near-α titanium alloys, spark plasma sintering, sintering temperature, microstructure, mechanical property

## Abstract

A near-α titanium alloy was fabricated using spark plasma sintering (SPS) to investigate the effects of sintering temperature on its relative density, microstructure, and mechanical properties. The relative density increased significantly with temperature, reaching 94.56%, 99.91%, and 99.99% at 850 °C, 900 °C, and 1000 °C, respectively. At 850 °C, the alloy contained numerous pores, leading to low density, while at 900 °C, full densification was achieved, resulting in a bimodal microstructure comprising 20% primary α phase (average size: 2.74 μm) and 80% transformed β phase (average lamellar width: 0.88 μm). Nanoscale equiaxed α phase (375 nm) and dispersed nanoscale β phase (80 nm) were observed within the lamellar structure. A distinct L-phase interfacial layer (50–100 nm) was identified at the α/β interfaces with a specific orientation relationship. At 1000 °C, the microstructure transformed into a fully lamellar structure with wider lamellae (1.99 μm), but mechanical properties declined due to coarsening. The alloy sintered at 900 °C exhibited the best properties, with a tensile strength of 989 ± 10 MPa at room temperature and 632 ± 10 MPa at 600 °C, along with elongations of 9.2 ± 0.5% and 13.0 ± 0.5%, respectively. These results highlight the importance of optimizing sintering temperature to balance densification and microstructural refinement for enhanced mechanical performance.

## 1. Introduction

Spark plasma sintering (SPS) is a novel powder consolidation technique that applies specific sintering power and pressure to the sintered powder using upper and lower punches and energized electrodes [1]. Through processes of discharge activation, thermoplastic deformation, and cooling, SPS achieves the production of high-performance materials [2]. This technology finds extensive application in preparing various materials, including nanomaterials [3], gradient materials [4], amorphous materials [5], and high-strength structural materials [6]. Compared to traditional hot pressing and sintering methods, SPS’s unique heating method allows for heating rates of several hundred degrees Celsius per minute, significantly reducing material processing cycles and enhancing production efficiency [7]. Furthermore, the discharge effect during SPS sintering partially removes impurities and oxide layers from the powder surface, activating the sintering material and improving sintering performance [8].

Meanwhile, due to the rapid heating and cooling during SPS, the resulting materials typically possess fine microstructures, showcasing excellent mechanical properties [9,10]. For example, Kang et al. utilized SPS to prepare Ti64 alloy with an ultrafine α + β dual nanocrystalline structure, exhibiting a room temperature tensile strength exceeding 1200 MPa and a fracture strain of 19.5% [11]. Hussein et al. employed SPS to fabricate a nearly fully dense Ti-20Nb-13Zr alloy with a microstructure composed of nanoscale α-Ti and equiaxed β-Ti, achieving a Vickers hardness 660 [12]. More significantly, using SPS technology in producing titanium alloys can lead to cost reductions ranging from 20% to 50% compared to other preparation methodologies [13]. Therefore, due to its efficient processing, superior mechanical properties, and cost-effectiveness, SPS technology emerges as one of the most promising techniques for manufacturing high-performance titanium alloys [14].

With the rapid development of the aerospace industry in the 21st century, there has been an increasing demand for high-performance materials [15], especially for components operating under high-temperature conditions (exceeding 600 °C) [16,17,18], such as turbine engine discs and blades [19]. Near-α titanium alloys and metal intermetallic compounds like TiAl have demonstrated the potential to replace some nickel-based high-temperature alloys [20]. Near-α titanium alloys offer room-temperature plasticity and processability advantages, thus presenting broad prospects in the aerospace field [21]. Several countries have developed a range of near-α titanium alloys, including the UK’s IMI834 alloy [22], the US’s Ti-1100 alloy [23], and China’s Ti60 alloy [24], designed to withstand service temperatures up to 600 °C, thereby significantly expanding the application range of titanium alloys.

The Ti60 alloy is a near-α titanium alloy reinforced with a complex composition of Ti-Al-Sn-Zr-Mo-Nb-Ta-Si, which combines solid solution strengthening, fine grain strengthening, and dispersion strengthening, showcasing outstanding comprehensive performance [25]. Currently, the preparation techniques for near-α high-temperature titanium alloys like Ti60 mainly involve casting methods [26]. Research on utilizing SPS technology to prepare Ti60 high-temperature titanium alloys remains relatively limited, and there is still an incomplete understanding of the microstructure of Ti60 high-temperature titanium alloys synthesized through SPS. Given this, in this study, we employed SPS technology to fabricate Ti60 alloy, investigating the effects of different sintering temperatures on the microstructure and mechanical properties of Ti60 alloy. We explored the fracture behavior of Ti60 alloy at room and high temperatures, aiming to deepen our understanding of the characteristics of near-α high-temperature titanium alloys synthesized via SPS. Ti60 alloy fabricated by SPS technology can be used for aerospace engine blades, gas turbine components, and critical parts in high-temperature chemical processing equipment, meeting the demands of high-end manufacturing for lightweight, high-strength, and heat-resistant materials.

## 2. Materials and Methods

### 2.1. Material

**Figure 1:**
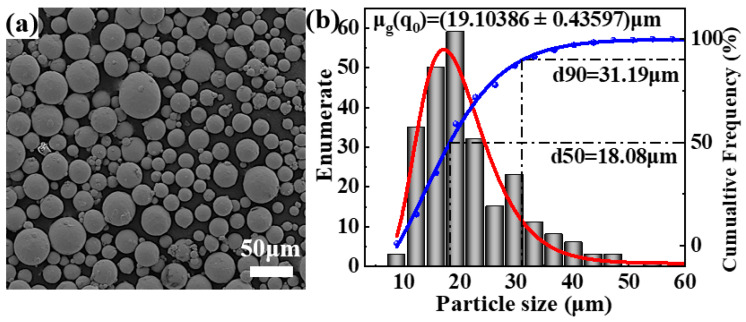
(**a**) Morphology of Ti60 alloy powder; (**b**) particle size distribution of Ti60 alloy powder (red: cumulative Frequency and (blue) enumerate fitting curve).

The Ti60 powder, prepared through gas atomization (Ti-5.8Al-4Sn-4Zr-0.7Nb-1.5Ta-0.4Si-0.06C), was procured from Asia New Materials (Beijing) Co., Ltd., Beijing, China. As depicted in Figure 1, the Ti60 alloy powder exhibits excellent sphericity, with an average particle size of 19.1 μm.

### 2.2. Preparation

Rapid consolidation experiments of the Ti60 alloy powder were conducted using a graphite die with an inner diameter of 20 mm in the SPS-3.20 equipment (Sojitz Machinery Corporation, Tokyo, Japan). Sintering temperatures were set at 850 °C, 900 °C, and 1000 °C, with a pressure of 50 MPa. The dwell time was set at 5 min, and the heating rate was maintained at 100 °C/min. Following SPS consolidation, the sample surfaces underwent grinding and polishing treatment. The samples were etched with Rolle reagent (85H_2_O:10HNO_3_:5HF) for 30–40 s.

### 2.3. Characterization

Density measurements of the samples were conducted using the Archimedes method, with each sample subjected to five measurements. The samples were machined into small cubes with dimensions of 8 × 8 × 8 mm, ensuring the absence of noticeable contaminants and oxide layers. Additionally, all samples underwent an impregnation treatment to ensure complete infiltration of deionized water into the pores. A high-precision electronic balance was used to measure the mass of the sample in air (*m*_1_). At 25 °C, the sample was fully immersed in deionized water, ensuring no air bubbles remained on the surface, and the mass under buoyant force (*m*_2_) was recorded using the electronic balance. According to Archimedes’ principle, the actual density (*ρ_p_*) of the material was calculated using Equation (1):

$$\rho_{p}=\frac{m_{1}}{m_{1}-m_{2}}\times \rho_{1}$$

where *ρ*_1_ is the density of deionized water. The crystal phases of the materials were identified using X-ray diffraction (XRD) analysis. The obtained diffraction patterns were compared with standard reference data from the JCPDS database to determine the phase composition. Additionally, selected-area electron diffraction (SAED) in TEM was used to further confirm the crystal structures of specific regions. The microstructure of the samples was observed using optical microscopy (OM), scanning electron microscopy (SEM, Hitachi S-4800N, Hitachi, Tokyo, Japan), and transmission electron microscopy (TEM, Talos F200X G2, Thermo Fisher Scientific, Waltham, MA, USA). The tensile properties of the samples at room temperature were evaluated using a universal testing machine equipped with a non-contact video extensometer (Zwick z2.5, ZwickRoell, Ulm, Germany). Tensile samples at 600 °C were tested using the LE5105 tensile machine (LISHI Instrument, Shang Hai, China) with a strain rate of 10^−3^ s^−1^ for both room temperature and 600 °C tests. Each sample was tested at least three times. Additionally, the fracture surfaces of the tensile specimens were observed using scanning electron microscopy.

## 3. Results and Discussion

### 3.1. Relative Density

Table 1 presents the relative densities of Ti60 samples prepared at three different sintering temperatures. The results indicate that as the sintering temperature increases, the relative density of the samples also increases, with the average relative density remaining above 90% for all three sintering temperatures. Specifically, the sample prepared at 850 °C exhibited the lowest relative density, measured at 95.46%. When the sintering temperature was increased to 900 °C, the relative density of the sample reached 99.91%, which is close to the theoretical density of Ti60 alloy. Further increasing the sintering temperature to 1000 °C resulted in a relative density of 99.99%, indicating complete densification of the alloy. These findings suggest that SPS can achieve high relative density of Ti60 alloy at relatively low temperatures, which can be attributed to the unique heating process and efficient thermal conductivity mechanisms of SPS

### 3.2. Microstructures

**Figure 2:**
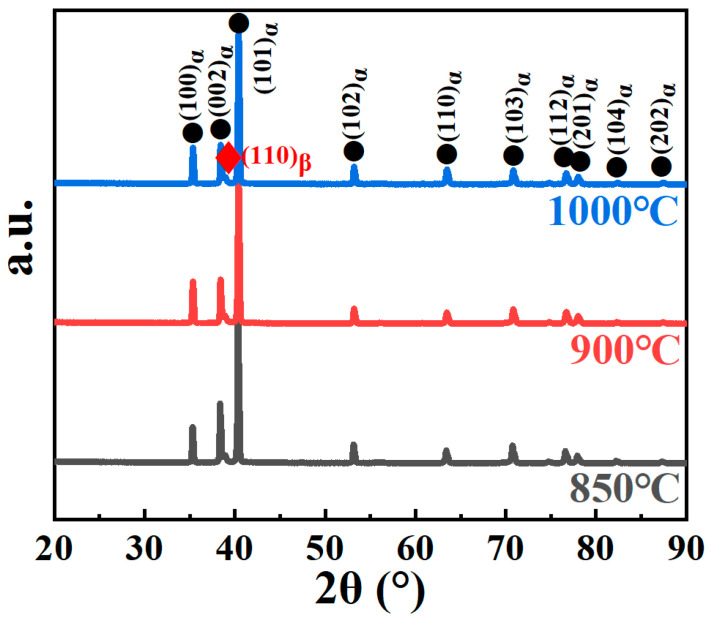
XRD patterns of samples produced at different temperatures.

The XRD patterns of Ti60 samples sintered at 850 °C, 900 °C, and 1000 °C are presented in Figure 2. As observed from the diffraction spectra, the primary diffraction peaks correspond to the α-Ti phase, indicating that the microstructure is predominantly composed of the hexagonal close-packed (HCP) α phase. Additionally, the diffraction peak corresponding to the (110) plane of the β phase is also detected, suggesting the presence of a retained body-centered cubic (BCC) β phase in the sintered samples. Notably, as the sintering temperature increases, the intensity of the β (110) diffraction peak exhibits a slight enhancement.

**Figure 3:**
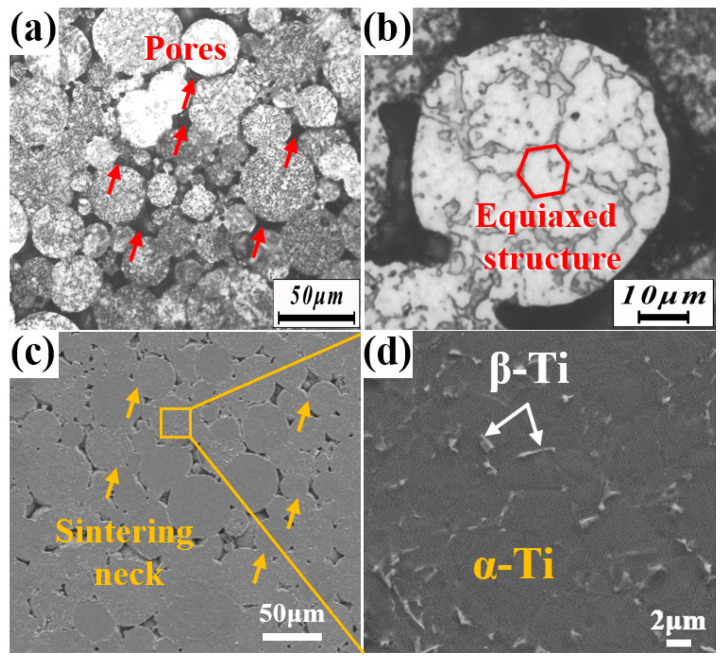
Microstructure of Ti60 alloy prepared at 850 °C sintering temperature: (**a**,**b**) OM and (**c**,**d**) SEM.

Figure 3 presents the microstructure of Ti60 alloy prepared at 850 °C. As shown in Figure 3a, although the relative density of the sample reached 94.56%, approximately 6% of internal porosity remains, and the powder particles have not fully metallurgically bonded, with their original spherical boundaries still clearly visible. Figure 3b shows that after sintering at 850 °C, the sample exhibits a dual-phase structure consisting of α and β phases, where the α phase is equiaxed, and the β phase is distributed between α phases. The SEM image in Figure 3c further indicates that the particles are connected by sintering necks. The higher magnification image in Figure 3d reveals that the gray area is the α phase, while the light gray area is the β phase.

**Figure 4:**
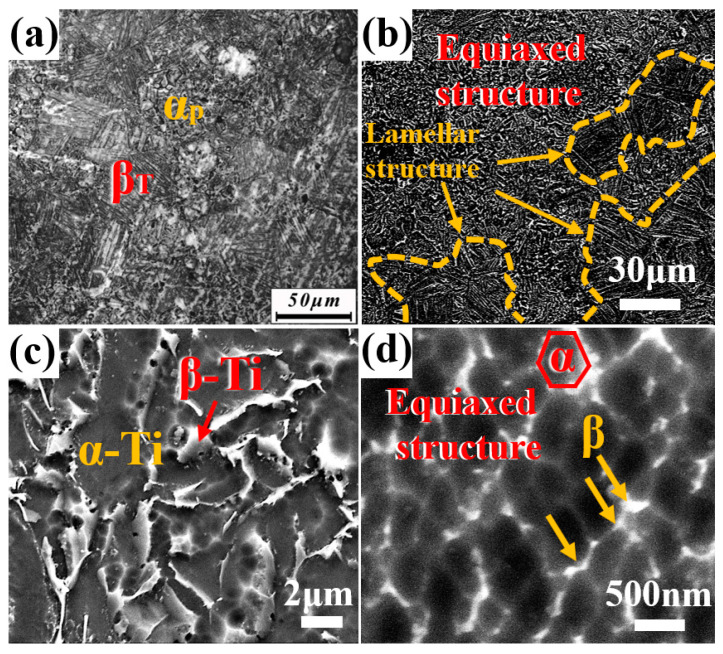
Microstructure of Ti60 alloy prepared at 900 °C sintering temperature: (**a**) OM and (**b**–**d**) SEM.

**Figure 5:**
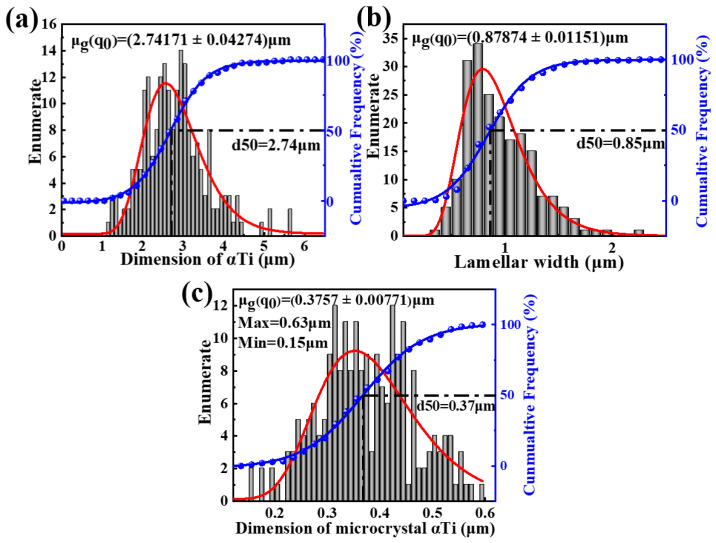
Size statistics of equiaxial α phase (**a**), layer width (**b**) and superfine α phase (**c**) in Ti60 alloy sintered at 900 °C.

Figure 4 presents the microstructure of Ti60 alloy sintered at 900 °C. As shown in Figure 4a, the microstructure of the sample exhibits a bimodal structure consisting of equiaxed and lamellar phases. Figure 4b displays the SEM image of the sample, where the gray region represents the α phase and the bright region represents the β phase. The internal porosity of the alloy has completely disappeared, indicating that metallurgical bonding between the powder particles has been successfully achieved at this sintering temperature. Figure 4c shows a higher magnification SEM image of the equiaxed region, where the size of the intergranular β phase ranges from 1 μm to 3 μm. The size of the β phase is significantly larger compared to the sample sintered at 850 °C, due to the higher sintering temperature. The statistical results in Figure 5a,b show that the average size of the equiaxed structure is approximately 2.74 μm, while the average width of the lamellar structure is approximately 0.88 μm. Image Pro Plus 6.0 software was used to calculate the proportion of equiaxed α phase, which is approximately 20%. The literature suggests that when the proportion of primary α phase in near-α titanium alloys is between 15% and 20%, the material typically exhibits superior mechanical properties [26]. Figure 4d presents a local magnification of the lamellar region, revealing that the lamellar structure is not composed solely of α phase, but rather consists of ultrafine equiaxed α phase and nanoscale β phase. This is in contrast to Ti60 alloys produced by conventional casting methods. The statistical results shown in Figure 5c indicate that the size distribution of the ultrafine equiaxed α phase ranges from 150 nm to 630 nm. Studies suggest that this α + β bimodal microcrystalline structure is formed during the SPS process, facilitated by forging stresses, secondary crystal slip/shear stresses, and a cooling rate of 200 °C/min during sintering [11].

**Figure 6:**
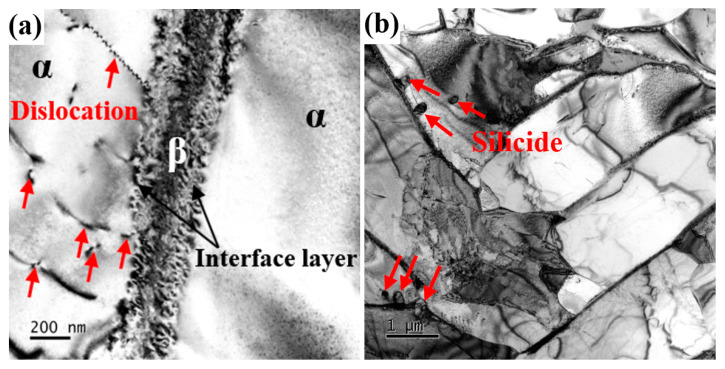
TEM images of Ti60 alloy sintered at 900 °C: (**a**) bright field image of α/β phase boundary and (**b**) bright field image of silicide.

**Figure 7:**
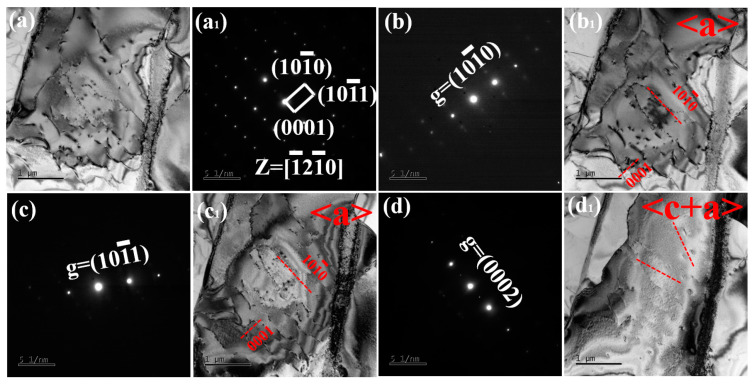
Dislocation structures of Ti60 alloy sintered at 900 °C: (**a**) BF-TEM image under Z = [$\bar{1}2\bar{1}0$], (**a_1_**) Diffraction pattern under Z = [$\bar{1}2\bar{1}0$], (**b**,**b_1_**) BF-TEM images of a grain taken under Z = [$\bar{1}2\bar{1}0$] zone axis with g = ($10\bar{1}0$) reflection, (**c**,**c_1_**) BF-TEM images of a grain taken under Z = [$\bar{1}2\bar{1}0$] zone axis with g = ($10\bar{1}1$) reflection, and (**d**,**d_1_**) BF-TEM images of a grain taken under Z = [$\bar{1}2\bar{1}0$] zone axis with g = (0001) reflection.

Figure 6 presents the TEM bright-field images of Ti60 alloy sintered at 900 °C. In Figure 6a, symmetrical interface layers are observed on both sides of the β phase, with a width of approximately 50–100 nm. The structure of these interface layers will be discussed in detail later. The dislocations in the α phase are evenly distributed, which contributes to enhancing the tensile strength of the Ti60 alloy. In Figure 6b, precipitates of silicides are observed at the α/β interface, with sizes ranging from 40 to 80 nm. During high-temperature tensile testing, these silicides effectively pin the α/β interface, playing a critical role in the alloy’s performance. Additionally, as shown in Figure 7a, a certain number of dislocations are observed within the α phase. These dislocations are generated during the sintering process to accommodate the deformation of the alloy, and can be referred to as geometrically necessary dislocations (GNDs). Figure 7b shows the diffraction pattern of the α phase along the [$\bar{1}2\bar{1}0$] axis. Figure 7(b_1_) and Figure 7(c_1_) present bright-field images taken under g = ($10\bar{1}0$) and g = ($10\bar{1}1$) reflections, respectively. It can be seen that these GNDs are almost all <a> dislocations. Due to the c/a ratio of α-Ti being 1.58, these dislocations are primarily activated by the prismatic slip system in the matrix. These GNDs generate significant backstress within the crystal, thereby enhancing the strength of the alloy.

**Figure 8:**
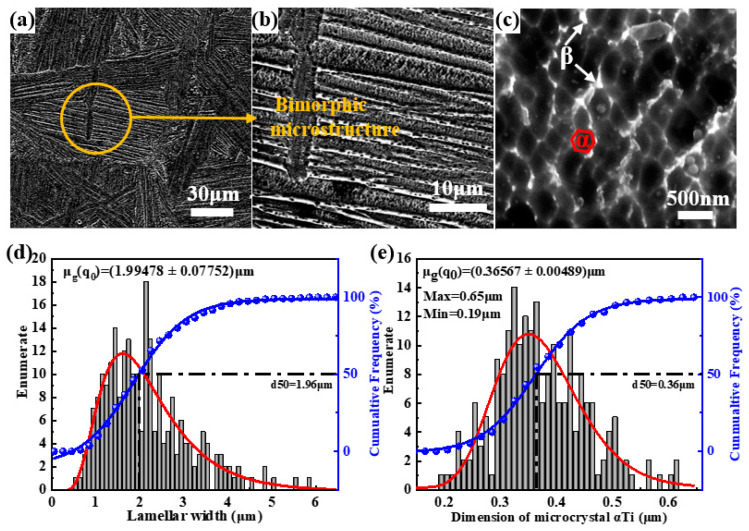
Microstructure and size statistics of Ti60 alloy sintered at 1000 °C: (**a**–**c**) SEM image, (**d**) size statistics of layer width and (**e**) size statistics of superfine α phase.

Figure 8 presents the microstructure of the Ti60 alloy sintered at 1000 °C. As shown in Figure 8a, the sample exhibits a fully lamellar structure, indicating that the sintering temperature has exceeded the β-transus temperature. Figure 8b,c show that, unlike the traditional Widmanstätten structure, the lamellar structure of the alloy is also composed of α + β dual microcrystals. In Figure 8d, it can be observed that as the sintering temperature increases from 900 °C to 1000 °C, the width of the lamellar structure within the Ti60 alloy increases from 0.88 μm to 1.99 μm, suggesting that high-temperature sintering promotes the development and enlargement of the lamellar structure. Figure 8e compares the Ti60 alloys sintered at 1000 °C and 900 °C, revealing that the size of the ultrafine equiaxed α phase within the lamellar structure shows no significant change, with the size range remaining between 190 nm and 650 nm. This indicates that although the higher sintering temperature facilitates the growth of the lamellar structure, the size of the ultrafine equiaxed α phase remains stable within this temperature range.

**Figure 9:**
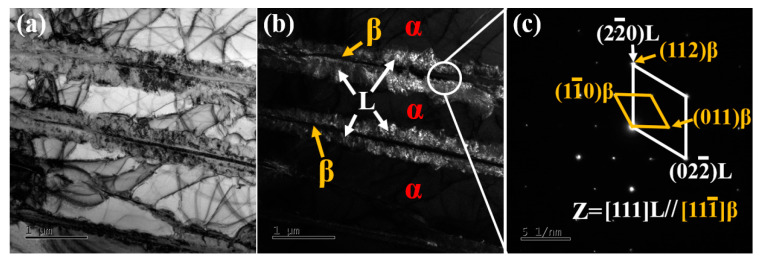
TEM images of Ti60 alloy sintered at 1000 °C: (**a**) bright field image, (**b**) dark field image, and (**c**) SAED pattern.

Figure 9 presents the TEM results of the Ti60 alloy sintered at 1000 °C. As shown in Figure 9a,b, symmetric interface layers are observed on both sides of the β phase, which are more clearly visible under dark field imaging. The average width of the interface layer is approximately 350 nm, which is wider than that observed in samples sintered at 900 °C. The diffraction patterns in Figure 9c show two sets of diffraction spots corresponding to the β and L phases, with their orientation relationship described by (112)_β_//($2\bar{2}0$)_L_ and [$11\bar{1}$]_β_//[111]_L_. The L phase, with a fcc crystal structure (a = 0.441 nm) [27], forms primarily due to solute segregation at the β phase interface during cooling, and the faster cooling rate in the SPS process promotes the formation of the L phase [28]. During tensile deformation, the L phase can effectively store dislocations, thereby improving the strength and ductility of the Ti60 alloy [28].

### 3.3. Mechanical Properties

**Figure 10:**
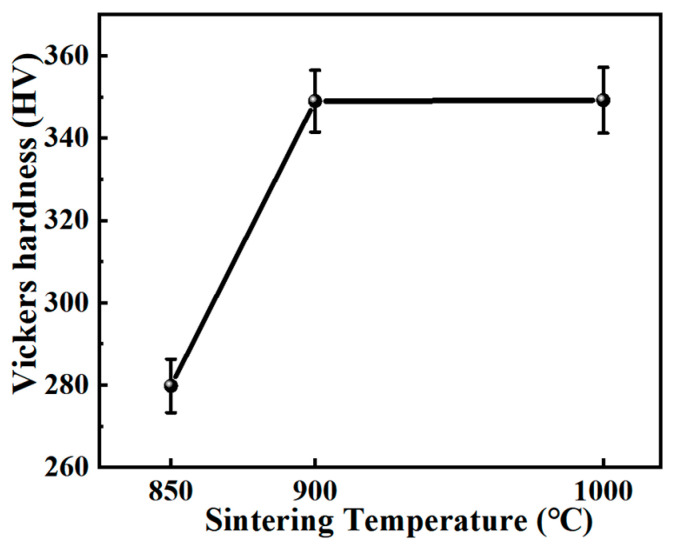
The Vickers hardness of Ti60 alloy prepared at different sintering temperatures.

Figure 10 presents the Vickers hardness test results of Ti60 alloy fabricated at different sintering temperatures. The results indicate that the relative density of the alloy has a significant impact on its Vickers hardness value. The sample sintered at 850 °C exhibited a Vickers hardness of 279.8 ± 7.0 HV, which is attributed to the presence of numerous internal pores, leading to a relatively low hardness value. As the sintering temperature was increased to 900 °C, the sample achieved full densification, and the Vickers hardness increased significantly to 349 ± 7.5 HV, representing a 24.7% improvement compared to the Ti60 alloy sintered at 850 °C. However, when the sintering temperature was further raised to 1000 °C, the Vickers hardness remained almost unchanged, indicating that the structural changes at this temperature had a minimal effect on the hardness.

**Figure 11:**
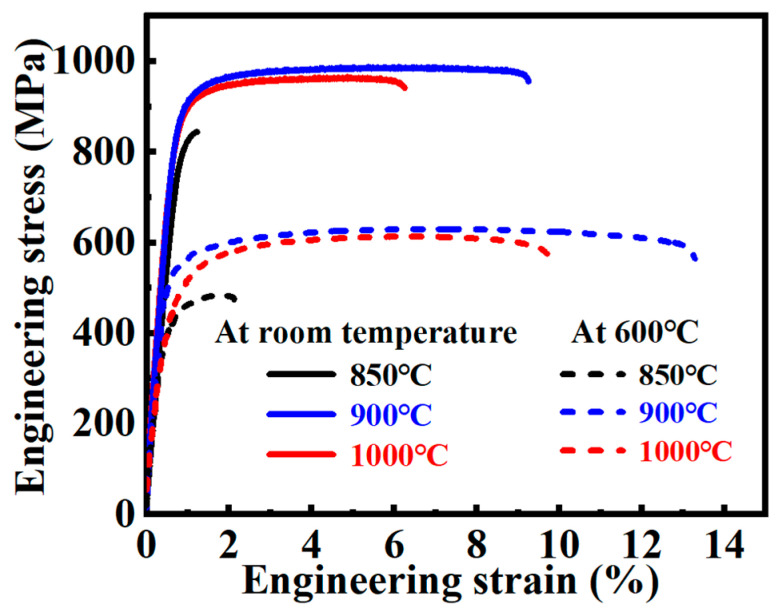
Quasi-static tensile curve of Ti60 alloy at room temperature and 600 °C.

Figure 11 shows the room-temperature and high-temperature quasi-static tensile engineering stress–strain curves of Ti60 alloys sintered at different temperatures. As the sintering temperature increases, the strength and ductility of the samples exhibit a trend of initially increasing and then decreasing. This phenomenon indicates that the sintering temperature has a significant impact on the mechanical properties of Ti60 alloys. For the sample sintered at 850 °C, the presence of internal porosity resulted in lower strength and ductility during both room-temperature and high-temperature tensile testing, exhibiting the poorest mechanical performance. The porosity not only reduced the effective load-bearing capacity of the alloy but also caused local stress concentration, which served as a crack initiation site, significantly affecting the alloy’s mechanical properties. As the sintering temperature was increased to 900 °C, the microstructure of Ti60 alloy improved, with complete elimination of internal porosity and successful metallurgical bonding of the powder particles, leading to optimal tensile performance at both room and high temperatures. Compared to the 850 °C sintered sample, the fracture elongation of the 900 °C sintered sample increased by 6.67 times at room temperature and 5.19 times at high temperature. This indicates that, at a sintering temperature of 900 °C, Ti60 alloy not only exhibited enhanced strength but also significant improvements in ductility. The increased ductility, particularly at high temperature, may be attributed to the synergistic effect between the primary α phase and β phase in the microstructure. However, when the sintering temperature was further increased to 1000 °C, both the room-temperature and high-temperature strength and ductility of Ti60 alloys decreased. This suggests that at higher sintering temperatures, grain coarsening in the alloy microstructure negatively affects its mechanical performance. The formation of a lamellar structure at this temperature resulted in a reduction in both strength and ductility. Additionally, the area enclosed by the stress–strain curve obtained from quasi-static tensile testing represents the material’s static toughness, with a larger area indicating better toughness. As shown in Figure 11, the Ti60 alloy sintered at 900 °C exhibits the highest toughness, while the alloy sintered at 850 °C demonstrates relatively poor toughness. This reduced toughness can be primarily attributed to the presence of a significant amount of internal porosity, which acts as stress concentration sites and weakens the material’s ability to resist fracture.

Overall, the Ti60 alloy sintered at 900 °C exhibited the best combination of strength and ductility, with the primary strengthening mechanisms including solid solution strengthening, dislocation strengthening, nanometer-scale silicide strengthening, and interface L-phase strengthening. These strengthening mechanisms worked synergistically during both room-temperature and high-temperature tensile tests, significantly enhancing the mechanical properties of the Ti60 alloy. Table 2 summarizes the room-temperature and high-temperature tensile properties of Ti60 alloys prepared at different sintering temperatures.

### 3.4. Fracture

**Figure 12:**
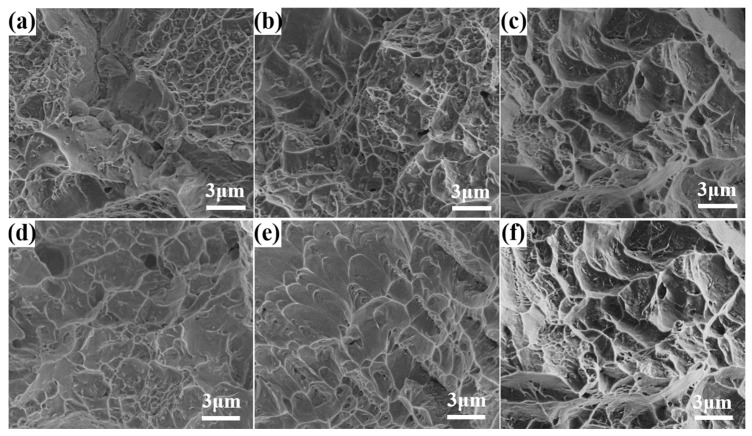
Room temperature and high temperature tensile fracture morphology of Ti60 alloy prepared at different sintering temperatures: (**a**–**c**) at room temperature and (**d**–**f**) at 600 °C.

Figure 12 presents the fracture morphology of Ti60 alloy specimens prepared at different sintering temperatures after tensile testing at both room temperature and high temperature. The microstructural evolution of the fracture surfaces provides critical insights into the failure mechanisms associated with different sintering conditions. As shown in Figure 12a, the fracture surface of the specimen sintered at 850 °C still retains the original morphology of the powder particles after tensile testing. The sintering necks formed during the SPS process exhibit insufficient bonding, which cannot effectively withstand high stress, leading to premature failure. Additionally, a significant number of residual pores are observed on the fracture surface, which act as stress concentration sites and significantly reduce the material’s ability to deform plastically, resulting in a low fracture elongation ratio. The presence of these defects suggests that the sintering temperature of 850 °C is not sufficient to achieve full densification and optimal mechanical properties. In contrast, as seen in Figure 12b,c, the fracture surfaces of specimens sintered at 900 °C and 1000 °C exhibit a more fully developed metallurgical bonding, with the disappearance of original powder particle boundaries. This indicates that a higher sintering temperature promotes diffusion and densification, leading to improved interparticle bonding and enhanced mechanical properties. The fracture morphology reveals a mixed fracture mode, characterized by both plastic deformation features and ductile dimples, suggesting a balance between strength and toughness. This improved bonding contributes to better mechanical performance, as the material can accommodate more plastic deformation before failure. Figure 12d shows the fracture surface of the specimen sintered at 850 °C after tensile testing at 600 °C, where brittle fracture characteristics are predominant. The insufficient bonding observed at this sintering temperature results in a material with limited ability to accommodate high-temperature deformation, making it more prone to brittle failure. The fracture surface exhibits cleavage-like features, further confirming its brittle nature under elevated temperatures. Figure 12e,f provide a comparison between fracture morphologies at room temperature and 600 °C. At 600 °C, a greater number of ductile dimples are observed, indicating improved plasticity at higher temperatures. This suggests that the elevated testing temperature enhances the mobility of dislocations and promotes plastic deformation, leading to increased ductility. The higher temperature likely reduces internal stresses and facilitates slip processes, thereby improving the fracture resistance of the material.

Overall, the fracture morphology analysis highlights the crucial role of sintering temperature in determining the mechanical performance of Ti60 alloys. While a sintering temperature of 850 °C results in incomplete bonding and higher porosity, leading to lower strength and ductility, increasing the sintering temperature to 900 °C or higher significantly enhances densification, metallurgical bonding, and overall mechanical properties. Additionally, high-temperature tensile testing reveals that ductility improves at elevated temperatures, further supporting the potential application of these alloys in high-temperature environments.

## 4. Conclusions

SPS-prepared Ti60 alloy and the influence of sintering temperature on its microstructure and mechanical properties were investigated, yielding the following conclusions:

(1) With the increase in sintering temperature, the relative density of Ti60 alloy first increased and then tended to stabilize. After sintering at 850 °C, 900 °C, and 1000 °C, the relative densities of Ti60 alloy were 94.56%, 99.91%, and 99.99%, respectively.
(2) At a sintering temperature of 900 °C, the microstructure of Ti60 alloy exhibits a bimodal characteristic, with approximately 20% equiaxed α phase, having an average size of 2.74 μm. The average width of the lamellar structure is 0.88 μm. Furthermore, within the lamellar structure, ultrafine equiaxed α phase and dispersed nanoscale β phase are observed, with average sizes of 375 nm and 80 nm, respectively.
(3) When the sintering temperature is increased to 1000 °C, the Ti60 alloy exhibits a fully lamellar microstructure, with the lamellar width increasing to approximately 1.99 μm. Additionally, both the 900 °C and 1000 °C sintered Ti60 alloys display a symmetrically distributed L-phase interface layer at the α/β interface, with an orientation relationship described by (112)_β_//($2\bar{2}0$)_L_ and [$11\bar{1}$]_β_//[111]_L_.
(4) The Ti60 alloy sintered at 900 °C exhibits the best mechanical properties. Its Vickers hardness is 349 ± 7.5 HV, with a room-temperature yield strength, tensile strength, and elongation of 887 ± 15 MPa, 989 ± 10 MPa, and 9.2 ± 0.5%, respectively. At 600 °C, the yield strength, tensile strength, and elongation are 535 ± 15 MPa, 632 ± 10 MPa, and 13.0 ± 0.5%, respectively.

## Figures and Tables

**Table 1:** Relative density of Ti60 alloy at different sintering temperatures.

Sintering Temperature	Relative Density
850 °C	94.56%
950 °C	99.91%
1000 °C	99.99%

**Table 2:** Room-temperature and high-temperature tensile properties of Ti60 alloy prepared at different sintering temperatures.

Material	Sintering Temperature	Test Temperature	YS (MPa)	UTS (MPa)	Elongation(%)
Ti60	850 °C	25 °C	816 ± 15	846 ± 20	1.2 ± 0.5
		600 °C	424 ± 10	483 ± 15	2.1 ± 0.5
	900 °C	25 °C	887 ± 15	989 ± 10	9.2 ± 0.5
		600 °C	535 ± 15	632 ± 10	13.0 ± 0.5
	1000 °C	25 °C	878 ± 10	962 ± 10	6.1 ± 0.5
		600 °C	511 ± 10	614 ± 10	9.6 ± 0.5

## Data Availability

The raw data required to reproduce these findings cannot be shared at this time, as the data also form part of an ongoing study.
